# Mutated *Rnf43* Aggravates *Helicobacter Pylori*-Induced Gastric Pathology

**DOI:** 10.3390/cancers11030372

**Published:** 2019-03-16

**Authors:** Victoria Neumeyer, Michael Vieth, Markus Gerhard, Raquel Mejías-Luque

**Affiliations:** 1 Institut für Medizinische Mikrobiologie, Immunologie und Hygiene, Technische Universität München, 81675 Munich, Germany; victoria.neumeyer@tum.de; 2 Institut für Pathologie, Klinikum Bayreuth, 95445 Bayreuth, Germany; vieth.lkpathol@uni-bayreuth.de

**Keywords:** RNF43, *H. pylori*, gastric pathology

## Abstract

The E3 ubiquitin ligase ring finger protein 43 (RNF43) is frequently mutated in gastric tumors and loss of RNF43 expression was suggested to be one of the key events during the transition from adenoma to gastric carcinoma. Functional studies on RNF43 have shown that it acts as a tumor suppressor by negatively regulating Wnt signaling. Interestingly, we observed that RNF43^H292R/H295R^ mice bearing two point mutations in the ring domain displayed thickening of the mucosa at early age but did not develop neoplasia. In this study, we infected these mice for 6 months with *Helicobacter pylori*, which has been described as one of the major risk factors for gastric cancer. Mice bearing mutant RNF43^H292R/H295R^ showed higher gastritis scores upon *H. pylori* infection compared to wild-type mice, accompanied by increased lymphocyte infiltration and *Ifng* levels. Furthermore, infected *Rnf43* mutant mice developed atrophy, hyperplasia and MUC2 expressing metaplasia and displayed higher levels of the gastric stem cell marker CD44 and canonical NF-κB signaling. In summary, our results show that transactivating mutations in the tumor suppressor *Rnf43* can worsen *H. pylori* induced pathology.

## 1. Introduction

Gastric cancer has been described as a multifactorial disease with high interindividual and intraindividual heterogeneity [1]. In recent years, much effort has been applied to define molecular signatures that may be useful in classifying and developing targeted therapeutic approaches for gastric cancer patients. To this end, analysis of the mutational landscape of gastric cancer has allowed generation of better molecular classifications and the definition of important genes driving gastric carcinogenesis. In this context, the E3 ubiquitin ligase ring finger protein 43 (RNF43) was found to be frequently mutated in gastric tumors [2,3,4] highlighting an important role for RNF43 in gastric carcinogenesis. RNF43 expression was shown to be lost in poorly differentiated gastric cancers [5], while mutations were concentrated in gastric carcinomas adjacent to adenomas [6], indicating an important role for RNF43 in the transition from adenoma to carcinoma. In addition, RNF43 downregulation in gastric tumors was associated with metastasis, TNM staging and poor survival [7], suggesting that loss of RNF43 function is one of the key events in gastric carcinogenesis. Our recent results using mice carrying a 57 bp deletion in exon 8 of *Rnf43* (*Rnf43^ΔEx8^*), confirmed an important role for RNF43 in gastric homeostasis, since mutant mice showed gastric hyperplasia, although lesions did not develop to neoplasia [8]. 

The tumor suppressor function of RNF43 is based on its ability to inhibit Wnt signaling at the level of Frizzled (FZD) receptors [9,10] as well as its capacity to sequester TCF4 to the nuclear envelope thereby disrupting transcription of Wnt target genes [11]. Interestingly, introduction of two point mutations (RNF43^H292R/H295R^) not only abolished its inhibitory effect but rather transactivated Wnt signaling [10,11], suggesting that certain mutations can have a further deleterious effect by enhancing Wnt signaling. Indeed, we identified in patients some mutations having this Wnt-transactivating effect in colon cells [11]. Furthermore, in the murine stomach, the introduction of the two point mutations leading to the amino acid substitutions H292R/H295R in the ring domain of RNF43 led to marked thickening of the gastric mucosa, hyperplasia and cellular atypia at early ages [8]. Whether these pathological changes depend on alterations of Wnt signaling could not be determined. Therefore, the identification of other signaling pathways or targeted genes needs to be further addressed. 

Apart from mutations in important genes, one of the main risk factors for gastric cancer development is chronic infection with *H. pylori*, which is classified as a class I carcinogen [12]. *H. pylori* induces strong Th1 and Th17 inflammatory responses, but at the same time favors the expansion of regulatory T cells, which create an immunosuppressive environment, possibly contributing to gastric cancer development [13,14,15]. *H. pylori*-induced chronic gastritis may progress to gastric atrophy, metaplasia and eventually to gastric cancer over years, in a process defined as the Correa pathway [16]. During this process, *H. pylori* de-regulates several signaling pathways, including the canonical and non-canonical NF-κB pathway [17,18] and the EGFR signaling pathway [19,20], and also promotes a mutation-prone milieu through ROS production and subsequent induction of DNA damage [21]. In addition, *H. pylori* can target the gastric stem cell compartment, leading to aberrant epithelial cell proliferation, metaplasia, and altered differentiation. Specifically, *H. pylori* was reported to accelerate the proliferation and expansion of LGR5^+^ cells [22] as well as CD44^+^ cells [23], which constitute important populations of stem cells in the stomach. Notably, expression of RNF43 was exclusively found in LGR5 intestinal crypt stem cells [10], while overexpression of RNF43 in gastric cells led to decreased protein levels of LGR5 [7]. In addition, xenografts derived from gastric RNF43 knockdown cells showed enhanced expression of the stem cell markers SOX2 and CD44, correlating with enhanced tumor growth [8]. Although these observations suggest an important role for RNF43 in the control of gastrointestinal stem cell homeostasis, it is still unknown whether RNF43 is also expressed in stomach stem cells or to what extent it controls the expression of other gastric stem cell markers in vivo. 

Considering the important role of RNF43 in gastric homeostasis and the high frequency of mutations observed in gastric tumors, as well as the carcinogenic potential of *H. pylori* infection, in the present study we sought to investigate how chronic *H. pylori* infection would affect the onset and development of gastric pathology in mice carrying mutated *Rnf43*. Our results show that *H. pylori* infection worsens gastric pathology of RNF43^H292R/H295R^ mice and suggest that *RNF43* mutations in combination with sustained chronic inflammation contribute to the development of gastric malignancies. 

## 2. Results

### 2.1. RNF43^H292R/H295R^ Mice Show Enhanced H. pylori-Induced Gastritis Inflammation

**Figure 1:**
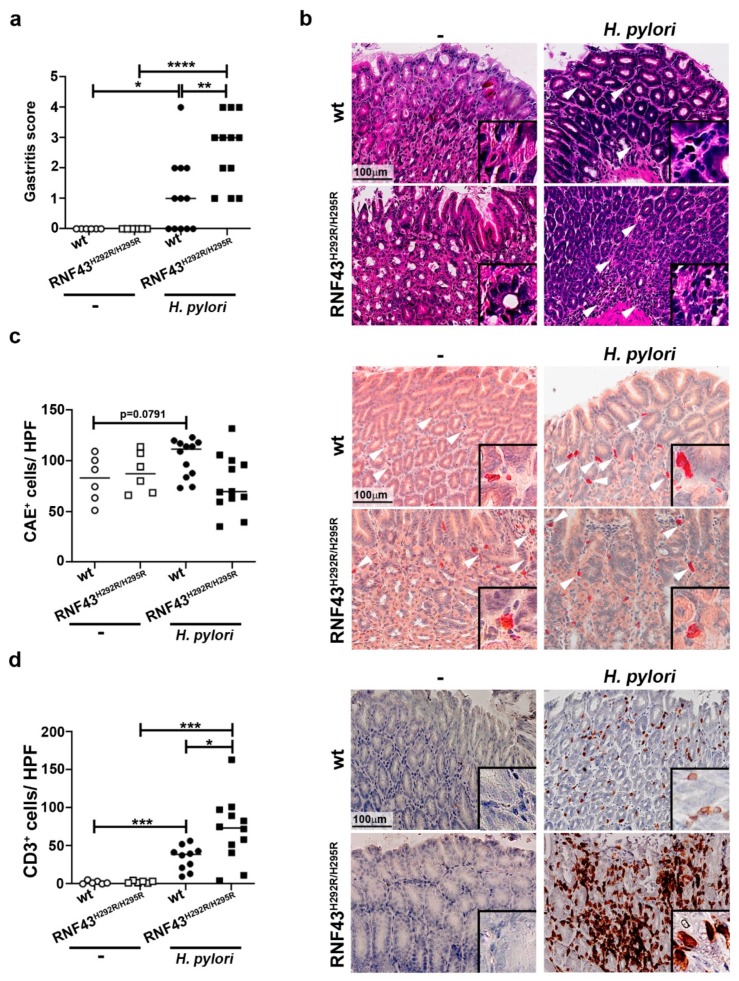

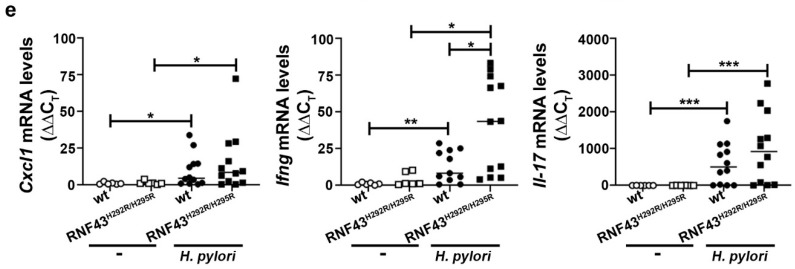
*H. pylori* infection worsens gastric inflammation of RNF43^H292R/H295R^ mice. (**a**) Gastritis score. Gastric inflammation was assessed according to the updated Sydney system for gastritis classification after 6 months of infection with the *H. pylori* strain PMSS1. (**b**) Representative images of H&E stained gastric sections showing infiltration of inflammatory cells (arrows) in the corpus of control and PMSS1 infected mice. (**c**) Quantification (positive cells per high power field (20× objective)) and representative images of chloracetate esterase (CAE) stained tissue samples. (**d**) Quantification of CD3^+^ cells per high power field and representative images of stained tissue samples. (**e**) mRNA levels of *Cxcl1*, *Ifng* and *Il-17* detected in gastric homogenates. Values were normalized to *Gapdh* and differences expressed as ΔΔC_T_ to control uninfected mice. * *p* ≤ 0.05; ** *p* ≤ 0.01; *** *p* ≤ 0.001; **** *p* ≤ 0.0001. Mann-Whitney Test (pairwise comparisons).

To analyze how the presence of *RNF43* transactivating mutations affects the inflammatory response to *H. pylori* chronic infection, we infected RNF43^H292R/H295R^ mice with the pathogenic *H. pylori* strain PMSS1 for six months. No significant differences in bacterial colonization were detected between wild-type and RNF43^H292R/H295R^ mice (Figure S1); however, RNF43^H292R/H295R^ mice showed higher inflammation scores in the stomach, evaluated according to the updated Sydney system for gastritis classification [24] (Figure 1a,b). Notably, no differences in neutrophil infiltration were detected between infected wild-type and RNF43^H292R/H295R^ mice at this point of chronic infection (Figure 1c). In contrast, lymphocytic infiltration, as detected by immunohistochemical staining of CD3^+^ cells, was higher in infected RNF43^H292R/H295R^ compared to infected wild-type mice (Figure 1d). 

To further characterize the immune response of RNF43^H292R/H295R^ mice compared to wild-type mice towards *H. pylori* infection, we analyzed the expression of different cytokines typically induced by *H. pylori* infection: *Cxcl1* (murine homologue of IL-8), as it relates to innate immunity, *Ifng* as a marker of Th1 responses, and *Il-17* as a marker of Th17 responses. *H. pylori*-infected wild-type mice showed increased expression levels of *Cxcl1* compared to uninfected mice (Figure 1e). Similar results were observed when comparing infected RNF43^H292R/H295R^ to uninfected RNF43^H292R/H295R^ mice, while no differences were detected between wild-type and RNF43^H292R/H295R^ mice upon infection (Figure 1e). As expected, *H. pylori* induced *Infg* expression in the stomachs of infected mice (Figure 1e). Interestingly, this expression was increased in infected RNF43^H292R/H295R^ mice, which showed higher levels of *Ifng* mRNA compared to infected wild-type mice (Figure 1e). *H. pylori* infection also induced IL-17 responses, as detected by high levels of *Il-17* expression upon infection (Figure 1e). However, no significant differences between wild-type and RNF43^H292R/H295R^ mice were observed (Figure 1e). 

Together these results suggest that transactivating mutations of *RNF43* aggravate gastric inflammation in response to *H. pylori*. 

### 2.2. H. pylori Increases Gastric Pathology of RNF43^H292R/H295R^ Mice

**Figure 2:**
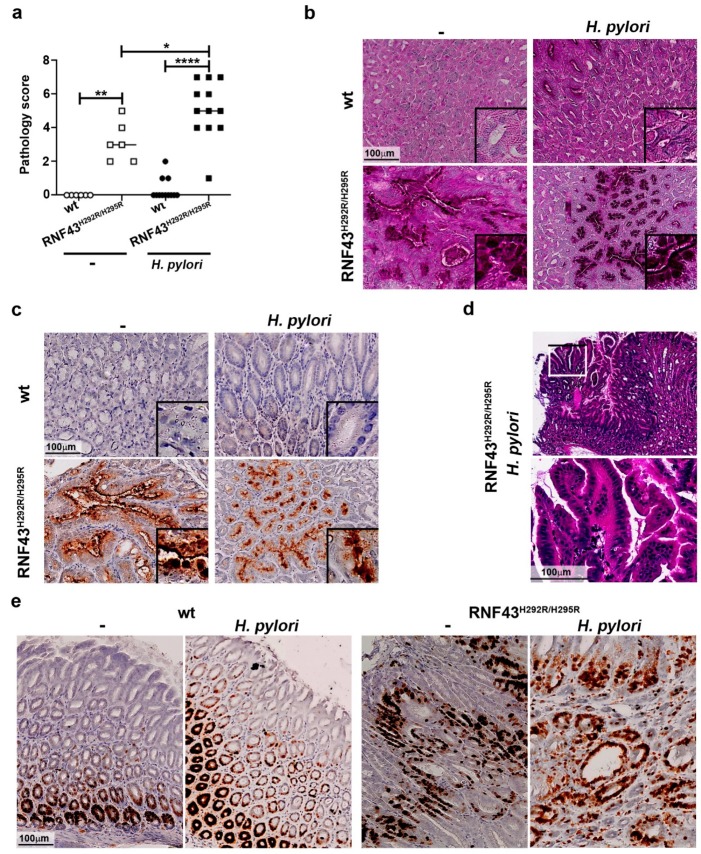
*H. pylori* enhances gastric pathology of RNF43^H292R/H295R^ mice. (**a**) Pathology score. The presence of atrophy, metaplasia, hyperplasia and reactive changes was assessed in antrum and corpus after 6-month *H. pylori* infection. (**b**) Representative images of periodic acid Schiff (PAS) stained gastric sections. (**c**) MUC2 expression detected in gastric tissue sections by immunohistochemistry. (**d**) Representative picture and higher magnification image of hyperplasia in the stomach of an infected RNF43^H292R/H295R^ mouse. * *p* ≤ 0.05; ** *p* ≤ 0.01; **** *p* ≤ 0.0001. Mann-Whitney Test (pairwise comparisons). (**e**) Ki67 staining denoting cellular proliferation in the stomach.

We have recently reported that RNF43^H292R/H295R^ mice show gastric hyperproliferation [8]. To explore whether *H. pylori* infection may influence gastric pathology in mice carrying a transactivating mutation of *Rnf43*, we analyzed the gastric mucosa of mice infected for six months. Macroscopically, we could detect lesions in the stomachs of *Rnf43* mutant mice, especially in the corpus, which were aggravated upon infection (Figure S2a). We established a pathology score based on the presence of atrophy, metaplasia, hyperplasia and reactive changes. Chronic *H. pylori* infection increased gastric pathology of RNF43^H292R/H295R^ mice (Figure 2a). Atrophy and metaplasia were detected only in the corpus of RNF43^H292R/H295R^ mice, under basal conditions as well as after *H. pylori* infection (Figure 2b). Metaplasia was observed to be multifocal in uninfected RNF43^H292R/H295R^ mice. Upon infection, metaplasia could be detected in most of the fields analyzed for some mice. Metaplasia was characterized by the expression of high levels of MUC2 (Figure 2c). Notably, no signs of atrophy or metaplasia were observed in the antrums of the mice (Figure S2b). Gastric pathology progressed to hyperplasia in 9 out of 12 (75%) of the infected RNF43^H292R/H295R^ mice, while 2 out 4 (50%) of RNF43^H292R/H295R^ mice showed hyperplasia without infection (Figure 2d). Hyperplasia was observed only in 2 out of 12 (17%) of infected wild-type mice. Reactive changes (nuclear enlargement, nuclear hyperchromasia, prominent nuclei and nuclear atypia) were detected in 25% of the uninfected RNF43^H292R/H295R^ mice. Upon *H. pylori* infection, 100% of the RNF43^H292R/H295R^ mice showed such reactive changes, while those were present in only 25% of the wild-type mice upon *H. pylori* infection. The lesions observed in *Rnf43* mutant mice were highly proliferative, as detected by Ki67 staining (Figure 2e). These results indicate that gastric pathology of RNF43^H292R/H295R^ mice is worsened by *H. pylori* infection. 

### 2.3. RNF43 Transactivating Mutations Favor the Expression of Genes Related to Gastric Carcinogenesis

**Figure 3:**
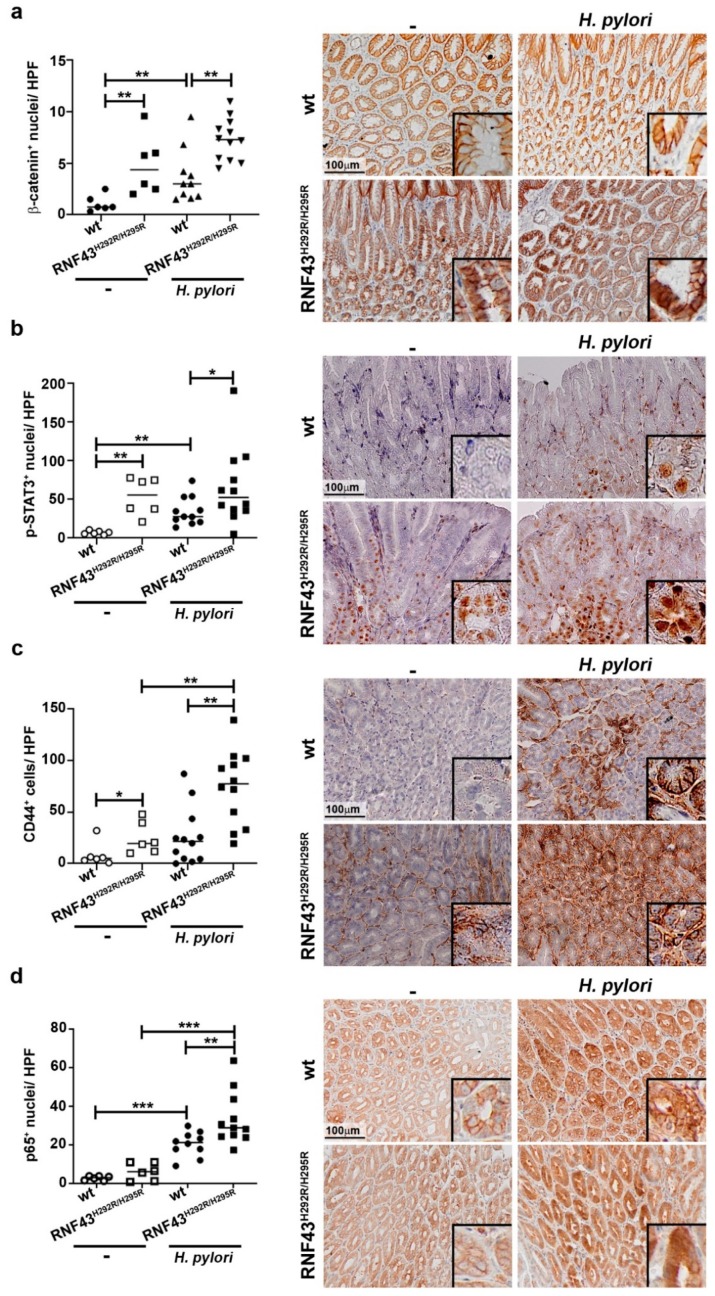
*H. pylori* enhances CD44 expression and NF-ĸB activation in the stomach of RNF43^H292R/H295R^. Number of β-catenin (**a**), p-STAT3 (**b**), CD44 (**c**) and nuclear p65^+^ cells (**d**) in the stomach of wild-type and RNF43^H292R/H295R^ mice under basal conditions and upon 6-month *H. pylori* infection. Representative images are shown. * *p* ≤ 0.05; ** *p* ≤ 0.01; *** *p* ≤ 0.001. Mann-Whitney Test (pairwise comparisons).

We analyzed the expression of genes related to the development of gastric tumors. We first studied the activation status of Wnt signaling by analyzing nuclear β-catenin. RNF43^H292R/H295R^ mice showed enhanced activation of the pathway compared to wild-type mice (Figure 3a). Notably, *H. pylori* infection led to increased nuclear translocation of β-catenin in wild-type as well as in RNF43^H292R/H295R^ mice (Figure 3a). 

Next, we analyzed the expression of *Sox2*, as previous studies have shown SOX2 to be deregulated during gastric carcinogenesis [25]. RNF43^H292R/H295R^ mice showed more expression of SOX2 in the stomach than wild-type mice (Figure S3a,b), although the difference was not significant. *H. pylori* infection slightly decreased SOX2 expression in wild-type mice, while infected RNF43^H292R/H295R^ mice showed markedly lower levels of SOX2 upon infection when compared to uninfected mutant mice (Figure S3a). 

STAT3 signaling has been linked to the development of gastric tumors in mice [26]. *H. pylori* infection induced phosphorylation of STAT3 in wild-type mice (Figure 3b). Under basal conditions, RNF43^H292R/H295R^ mice showed a higher number of p-STAT3^+^ cells compared to wild-type mice. Interestingly, *H. pylori* infection did not alter the phosphorylation of STAT3 in RNF43^H292R/H295R^ mice (Figure 3b), but RNF43^H292R/H295R^ mice presented a higher number of p-STAT3^+^ cells compared to infected wild-type mice (Figure 3b). These results indicate that changes in STAT3 signaling may contribute to the gastric pathology observed in RNF43^H292R/H295R^ mice, but not to the increased pathology driven by *H. pylori* infection. 

We also analyzed the expression of the stem cell marker CD44, which has been associated with initiation and progression of gastric cancer and previous studies have shown that CD44^+^ cells are targeted during *H. pylori* infection [27]. We observed a higher number of CD44^+^ cells in the stomach of uninfected RNF43^H292R/H295R^ mice compared to wild-type control mice (Figure 3c). *H. pylori* infection led to increased numbers of CD44^+^ cells in wild-type mice, although the differences were not statistically significant (Figure 3c). Notably, infected RNF43^H292R/H295R^ mice displayed much higher numbers of CD44^+^ cells in the gastric mucosa upon *H. pylori* infection compared to uninfected RNF43^H292R/H295R^ mice as well as infected wild-type mice (Figure 3c), suggesting *H. pylori*-targeted expansion of CD44^+^ gastric stem cells is enhanced in the presence of *RNF43* transactivating mutations. 

Finally, given the importance of NF-ĸB signaling during *H. pylori* infection and gastric cancer development, we analyzed activation of NF-ĸB in the stomach by assessing the number of cells presenting nuclear expression of p65. We observed that upon infection with *H. pylori*, NF-ĸB was activated in wild-type mice, as expected (Figure 3d). RNF43^H292R/H295R^ mice showed no basal activation of the pathway. Upon infection, RNF43^H292R/H295R^ mice showed enhanced p65 nuclear translocation in gastric cells compared to uninfected RNF43^H292R/H295R^ and infected wild-type mice. These observations suggest that NF-ĸB might be involved in the enhanced pathology observed in RNF43^H292R/H295R^ mice in response to *H. pylori* infection. In addition, we analyzed the expression of the canonical and non-canonical NF-ĸB target genes *Cxcl10* and *Cxcl13*, respectively. *Cxcl10* mRNA levels were higher in infected wild-type mice compared to uninfected mice (Figure S3c). Infected RNF43^H292R/H295R^ mice showed higher levels of *Cxcl10* compared to uninfected RNF43^H292R/H295R^ mice as well as to infected wild-type mice, confirming increased activation of canonical NF-ĸB. Although *H. pylori* induced the expression of *Cxcl13* in the stomach (Figure S3d), as we have previously reported [18], we could not detect differences between infected wild-type and RNF43^H292R/H295R^ mice, excluding the involvement of non-canonical NF-ĸB in the phenotype observed for infected RNF43^H292R/H295R^ mice. 

Together, our results indicate that increased pathology induced by *H. pylori* in the presence of *RNF43* transactivating mutations may be supported by increased expression of CD44 and hyperactivation of canonical NF-ĸB. 

## 3. Discussion

Gastric cancer is the fifth most frequently diagnosed cancer and the third leading cause of cancer related deaths worldwide [28]. Whole exome and whole genome sequencing studies reported *RNF43* to be frequently mutated in gastric tumors. *RNF43* mutations are more often found in microsatellite instable tumors [2,3,4] and truncating mutations are the most common type of mutations [3]. Using a model of CRISPR/Cas9 engineered mice, we found that the introduction of two point mutations in the ring domain of murine *Rnf43* did not impact intestinal homeostasis, but induced relevant changes in the stomach architecture of RNF43^H292R/H295R^ mutant mice [8]. Nevertheless, pathology did not progress to neoplasia, indicating that mutations in *RNF43* alone are not sufficient to drive malignant transformation. In the present study, we combined the presence of mutated *Rnf43* with chronic *H. pylori* infection, since the latter is a major risk factor for the development of gastric cancer. We observed that RNF43^H292R/H295R^ mice showed increased inflammation characterized by high lymphocytic infiltration and IFNγ production, and worsened pathology compared to uninfected RNF43^H292R/H295R^ and infected wild-type mice. This suggests that mutations in *RNF43* render the gastric mucosa more susceptible to *H. pylori* infection. 

When exploring possible mechanisms driving pathology in *Rnf43* mutant mice, we analyzed phosphorylation of STAT3, since this signaling pathway has been previously related to gastric carcinogenesis [26]. RNF43^H292R/H295R^ mice presented increased number of p-STAT3^+^ nuclei in the gastric mucosa compared to wild-type mice. However, *H. pylori* infection did not further enhance STAT3 activation, suggesting that STAT3 signaling may be involved in the pathology observed in *Rnf43* mutant mice already under basal conditions, and may favor enhanced pathology upon infection by crosstalk with other signaling pathways. 

We further observed that mice carrying mutated *Rnf43* showed enhanced nuclear accumulation of β-catenin in the stomach. Several components of the Wnt signaling pathway, including activators as well as suppressors, have been described to be de-regulated during gastric carcinogenesis (reviewed in [29]). The Wnt pathway plays a critical role in stem cell proliferation, but it also supports the development and renewal of cancer stem cells. Although the origin of gastric cancer stem cells is still unclear, it is suggested that they are responsible for the formation, maintenance, and continuous growth of the tumors [30,31,32]. Notably, gastric cancer stem cells are characterized by the expression of CD44 [33,34], which is a target gene of the Wnt signaling pathway [33]. We observed that RNF43^H292R/H295R^ mice showed higher levels of CD44 than wild-type mice under basal conditions. This could be related to a de-regulated Wnt pathway as a result of *Rnf43* mutation. Interestingly, *H. pylori* infection led to an even higher number of CD44^+^ cells in the stomach. *H. pylori* has been previously shown to target and expand the CD44^+^ gastric stem cell compartment [23], while inhibition of CD44 blocked proliferation and cancer progression in *H. pylori*-infected gerbils [27]. Further, experiments using *CD44*^−/−^ mice showed that CD44 is crucial for the development of mucous metaplasia during *H. pylori* infection [35]. In humans, progression of precancerous gastric lesions was associated with increased levels of CD44 in *H. pylori*-infected subjects [35]. In line with these observations, the expression of CD44 in the stomach of RNF43^H292R/H295R^ mice was related to the presence of metaplasia and progression of the gastric lesions, and both pathology and CD44 expression were associated with *H. pylori* infection. The expansion of CD44^+^ gastric cells in mice was suggested to be induced by a cooperative effect of PGE_2_-mediated inflammation and Wnt signaling, resulting in the development of gastric tumors [36]. In addition, Wnt-1 was related to CD44 expression [37], while inhibition of the Wnt/β-catenin pathway suppressed gastric cancer stem cells [37]. These observations confirmed an important role for Wnt signaling in the control of CD44^+^ gastric stem cells. However, *H. pylori* has been described to enhance Wnt signaling by inducing nuclear β-catenin accumulation independently of upstream components of the Wnt pathway [38,39,40]. Therefore, *H. pylori*-enhanced expansion of CD44^+^ cells observed in RNF43^H292R/H295R^ mice may be further regulated by an additional mechanism independent of Wnt. In fact, the expression of CD44 in different cancer cells has been reported to be regulated by NF-ĸB [41,42,43], a signaling pathway that is rapidly activated in gastric epithelial cells upon *H. pylori* infection [44]. Thus, NF-ĸB signaling is a major contributor to *Helicobacter*-induced gastric pathology and epithelial malignant transformation [45,46]. In light of our results, it is tempting to speculate that activation of NF-ĸB by the infection further up-regulates the expression of CD44, thereby inducing the progression of gastric lesions to more severe pathology. Nevertheless, it is still unclear why activation of NF-ĸB is higher in RNF43^H292R/H295R^ mice than in wild-type mice upon infection; thus, this deserves further investigation. 

Although mutations in *RNF43* seem to lead to a more severe pathology in response to *H. pylori* infection, it has not been addressed whether *H. pylori* may induce mutations in *RNF43*. To date, no correlations between *H. pylori* infection and *RNF43* mutation status in patients have been established. However, analysis of *RNF43* mutations during progression of tumors from low grade to high grade dysplasia and early gastric cancer revealed that mutations in *RNF43* occurred at the stage of high grade dysplasia and early gastric tumors [6]. This indicates that *H. pylori*-induced preneoplastic changes precede *RNF43* mutations. Considering the high prevalence of *H. pylori* infection and the increased incidence of *RNF43* mutations detected in gastric tumors, further studies should be conducted to understand how both events interrelate. 

Together, our results using a novel mouse model provide evidence that a chronic inflammatory milieu contributes to progression of gastric lesions when *RNF43* is mutated in gastric cells. This finding opens the pathway to further investigations in order to address whether *H. pylori* infection aggravates cancer progression in subjects carrying mutations in *RNF43*, as well as the molecular mechanisms engaged. 

## 4. Materials and Methods

### 4.1. Helicobacter Pylori Infection

RNF43^H292R/H295R^ mice were generated by introducing two point mutations in the RING domain of Rnf43 through homology directed repair, as described in [8]. 6–8-week old mice were infected twice with 2 × 10^8^ *H. pylori* strain PMSS1 [47] diluted in 200 µL brain-heart-infusion (BHI) containing 20% FCS by oral gavage and sacrificed after 6 months.

To this end, PMSS1 was cultured on Wilkins-Chalgren (WC) Dent agar plates in a microaerophilic atmosphere (5% O_2_, 10% CO_2_). For infection, bacteria were collected in BHI 20% FCS and density was measured at OD_600_ (OD_600_ 1 = 2 × 10^8^ bacteria/mL).

All animal experiments were conducted in compliance with European guidelines for the care and use of laboratory animals and were approved by the Bavarian Government (Regierung von Oberbayern, AZ.55.2-1-54-2532-196-2016).

### 4.2. Quantitative PCR

Murine stomach tissue pieces were homogenized with a Precellys lysing Kit and RNA was extracted using a Maxwell 16 LEV simply RNA Tissue Kit and a Maxwell 16 MDx Instrument (Promega, Madison, WI, USA) RNA was retrotranscribed using Moloney Murine Leukemia Virus Reverse Transcriptase RNase H- Point Mutant (Promega). GoTaq qPCR Mastermix (Promega) and a CFX384 system (Bio-Rad, Hercules, CA, USA) were used to assess transcript abundance with the recommended standard quantitative PCR cycling program. The PCR conditions used were 40 cycles of amplification with 15 s denaturation at 95 °C and 1 min annealing and amplification at 60 °C. Normalization to GAPDH and the comparative ΔΔCT method was used to analyze target gene expression. Primers used in this study are summarized in Table 1.

### 4.3. Immunohistochemistry

Mice were dissected and tissue was fixed in 4% formaldehyde and subsequently embedded in paraffin. For histologic evaluation, hematoxylin and eosin, chloroacetate esterase staining and periodic acid Schiff (PAS) staining were performed. For immunohistochemistry, 10 mM sodium citrate (pH 6) or 1 mM EDTA (pH 8) (p-STAT3) was used for heat induced antigen retrieval. Primary antibodies were applied overnight or for 1 h (MUC2) at room temperature (Table 2) and detected by horseradish peroxidase coupled secondary antibodies. Signal was detected using diaminobenzidine (DAB), and slides were scanned and analyzed using an Olympus Virtual Slide Imaging system (Olympus, Shinjuku, Japan).

To assess the pathology observed in the stomach a pathology score was established by assessing the degree of atrophy (0–3), the presence of metaplasia (0-absent, 1-multifocal, 2-in most fields, 3-widespread in all fields), the presence of hyperplasia (0-absent, 1-slight, 2-moderate, 3-severe), and reactive changes (0-absent, 1-present).

## 5. Conclusions

Mutation of *Rnf43* worsens *H. pylori*-induced pathology in the stomach. *Rnf43* mutations increase the levels of nuclear β-catenin and of phosphorylated STAT3 in the gastric mucosa, while *H. pylori* in addition enhances activation of NF-κB signaling and expression of the stem cell marker CD44. Thus, the activation of these different signaling pathways in concert seem to aggravate pathology in the stomach. This data provides functional hints for a novel role of *RNF43* mutations in gastric carcinogenesis.

## Figures and Tables

**Table 1:** Sequence of the primers used in this study.

Gene Name	Forward	Reverse
*Cxcl10*	AAGTGCTGCCGTCATTTTCT	CCTATGGCCCTCATTCTCAC
*Cxcl13*	ATATGTGTGAATCCTCGTGCCA	GGGAGTTGAAGACAGACTTTTGC
*Gapdh*	GCCTTCTCCATGGTGGTGAA	GCACAGTCAAGGCCGAGAAT
*Ifng*	TCAAGTGGCATAGATGTGGAAGAA	TGGCTCTGCAGGATTTTCATG
*Il-17*	GCTCCAGAAGGCCCTCAGA	AGCTTTCCCTCCGCATTGA
*Cxcl1*	TGCACCCAAACCGAAGTCAT	TTGTCAGAAGCCAGCGTTCAC
*Sox2*	CATGGGCTCTGTGGTCAAGT	CGGGGAGGTACATGCTGATC

**Table 2:** Antibodies used in this study.

Target	Clone	Company
CD3	SP7	Thermo Fisher
CD44	E7K2Y	Cell Signaling
MUC2	LUM 2.3	[48]
p-STAT3	D3A7	Cell Signaling
p65	D14E12	Cell Signaling
SOX2	C70B1	Cell Signaling

## Supplementary Materials

The following are available online at https://www.mdpi.com/2072-6694/11/3/372/s1, Figure S1: Colony forming units in the stomach of *H. pylori*-infected mice, Figure S2: Gastric pathology in RNF43^H292R/H295R^ mice is mainly observed in the corpus, Figure S3: Representative images and quantification of SOX2 expression (a) and mRNA expression levels of *Sox2* (b), *Cxcl10* (c) and *Cxcl13* (d) in the stomach of control and infected mice.
