# Supplementary material for: Mutated Rnf43 Aggravates Helicobacter Pylori-Induced Gastric Pathology
Source: Cancers (Basel). 2019 Mar 16;11(3):372. doi: 10.3390/cancers11030372 (PMC6468876; doi:10.3390/cancers11030372)
Supplement: Supplementary file 1 [file cancers-11-00372-s001.pdf]

Supplementary Materials

Mutated *Rnf43* Aggravates *Helicobacter Pylori*-Induced Gastric Pathology

Victoria Neumeyer, Michael Vieth, Markus Gerhard and Raquel Mejías-Luque

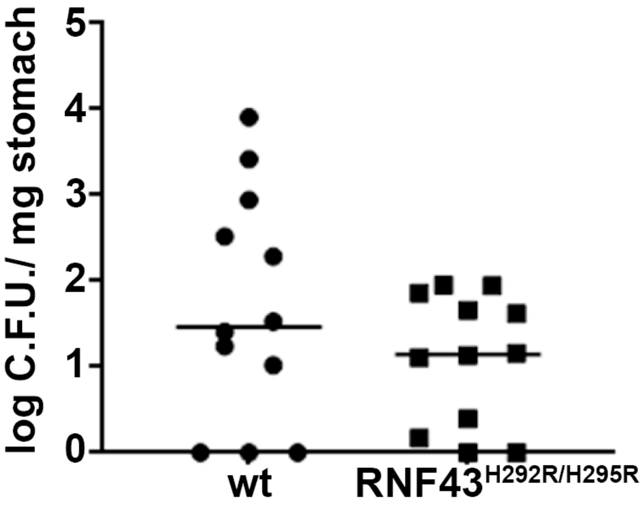

Figure S1. Colony forming units in the stomach of *H. pylori*-infected mice.

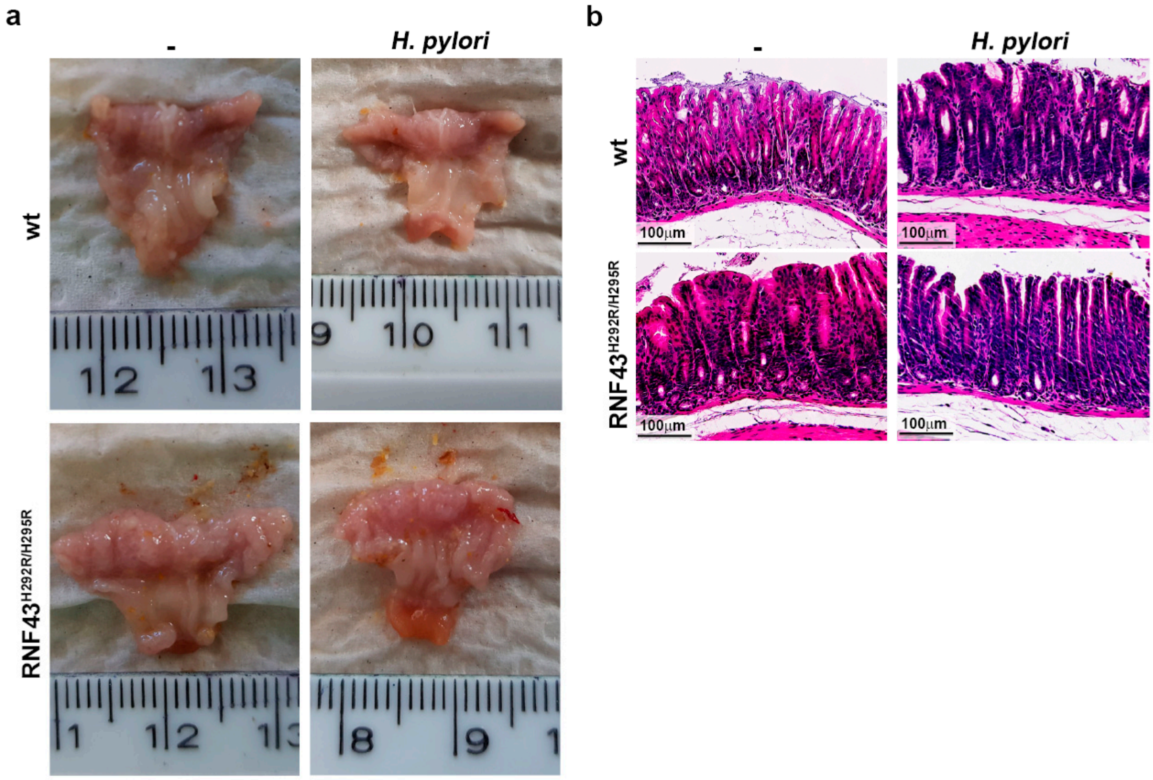

Figure S2. Gastric pathology in RNF43$^{H292R/H295R}$ mice is mainly observed in the corpus. (**a**) Macroscopic images of whole stomachs. (**b**) Representative H&E stainings of stomach antrum.

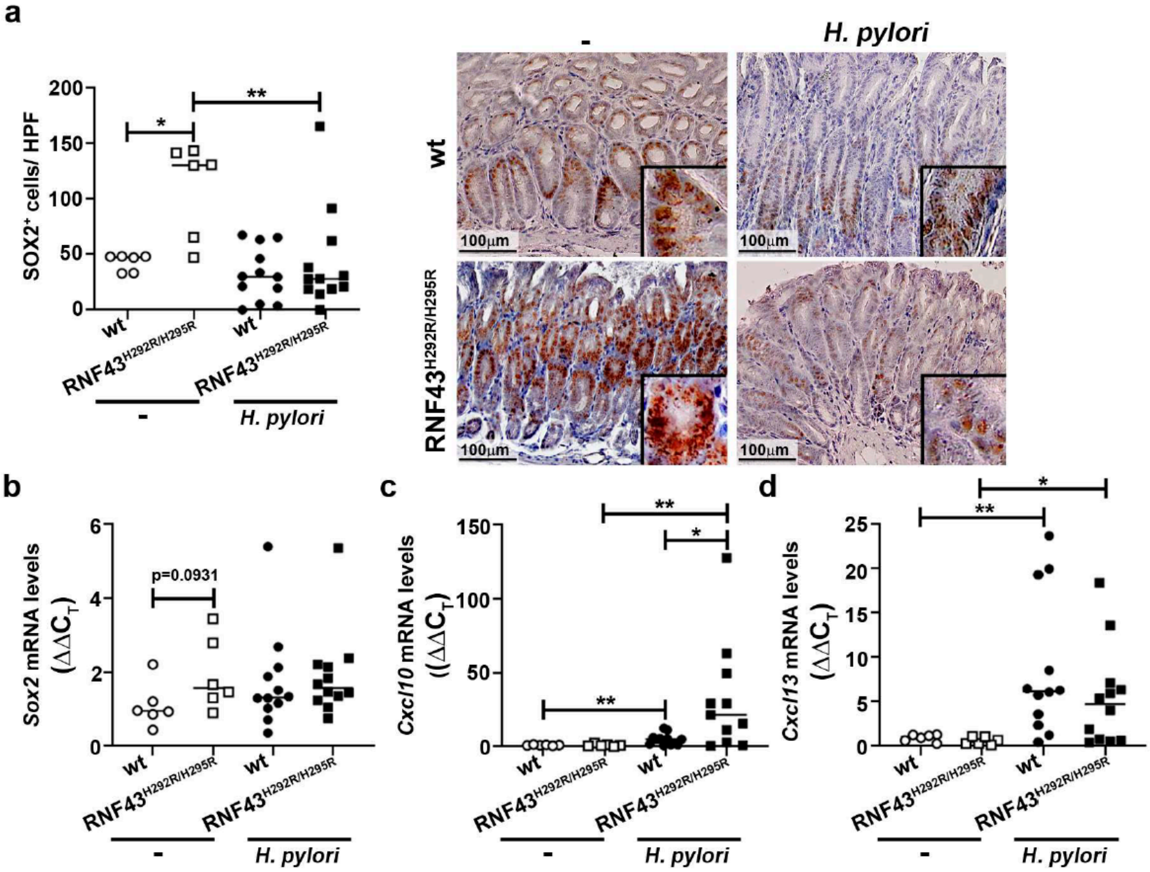

Figure S3. Representative images and quantification of SOX2 expression (**a**) and mRNA expression levels of *Sox2* (**b**), *Cxcl10* (**c**) and *Cxcl13* (**d**) in the stomach of control and infected mice.

© 2019 by the authors. Licensee MDPI, Basel, Switzerland. This article is an open access article distributed under the terms and conditions of the Creative Commons Attribution (CC BY) license (http://creativecommons.org/licenses/by/4.0/).
